# Metabolic enhancement of the one carbon metabolism (OCM) in bovine oocytes IVM increases the blastocyst rate: evidences for a OCM checkpoint

**DOI:** 10.1038/s41598-022-25083-8

**Published:** 2022-11-30

**Authors:** Arefeh Golestanfar, Amir Niasari-Naslaji, Farnoosh Jafarpour, Shiva Rouhollahi, Naeimeh Rezaei, Yves Menezo, Maurizio Dattilo, Mohammad Hossein Nasr-Esfahani

**Affiliations:** 1 Department of Theriogenology, Faculty of Veterinary Medicine, University of Tehran, Tehran, Iran; 2 Department of Animal Biotechnology, Reproductive Biomedicine Research Center, Royan Institute for Biotechnology, ACECR, Isfahan, Iran; 3 Laboratoire Clément, 17 Avenue d’Eylau, 75016 Paris, France; 4 Parthenogen, Lugano, Switzerland

**Keywords:** Embryology, Epigenetics

## Abstract

The one carbon metabolism (OCM) has a primary role in the process of oocyte maturation. In this study bovine oocytes were cultured for 24 h, up to MII stage, with standard medium supplemented or not with 8 metabolic enhancers of the OCM and the MII and blastocyst rate were compared. Additional analyses were performed on matured oocytes, cumulus cells, zygotes and blastocysts. The OCM supplementation increased the blastocyst rate derived from in vitro fertilization. The mitochondrial mass and DNMT3a protein expression were increased whereas DNA fragmentation decreased in matured oocytes. DNA methylation in female pronucleus of zygotes was increased. The supplementation did not directly affect the redox balance as ROS and GSH in matured oocytes and homocysteine in the spent medium were unchanged. The supplementation of the oocytes with metabolic enhancers of the OCM may increase the yield from the culture, likely due to improved DNA methylation and epigenetic programming. The lack of effects on MII rate with huge differences appearing at the blastocyst stage suggest the existence of a OCM metabolic check point that hampers oocytes progression to blastocyst post-fertilization, if they were not properly primed at the time of maturation.

## Introduction

In vitro embryo production is a valuable tool to assist childless couples and to propagate genetically modified livestock^1,2^. Recently, several investigations have focused on optimizing the in vitro culture media in order to make them closer to the in vivo physiological microenvironment “niche” of oocyte and/or embryo development^3–5^.

The oocyte is surrounded by a microenvironment influencing its metabolic status. Any perturbations of this metabolic status may hamper its developmental competence^6^. The basic metabolic pattern of mammalian oocytes and pre-implantation embryos, such as the consumption of oxygen and the utilization of nutrients, mainly glucose, pyruvate and lactate, is well characterized^7^. More recently, Li et al. investigated the dynamic metabolome of mouse oocytes during in vivo maturation by isolating a large number of oocytes at GV, MI and MII stages^8^. They found that, during meiotic resumption, the one carbon metabolism (OCM) was upregulated with increased amounts of various metabolites in the methionine cycle and in the trans-sulfuration pathway. OCM is the pathway that recycles homocysteine to methionine, which is crucial for cell growth, differentiation, epigenetic regulation (methylations), antioxidant cascade and energy balance^9^. The OCM consists of three related pathways: Folate and methionine cycles and the trans-sulfuration pathway^10^. In the methionine cycle, remethylation of homocysteine can be carried out via two alternative pathways: In the most ubiquitous pathway, the methyl group carried by 5-methyltetrahydrofolate (5-methyl-THF) is transferred to homocysteine by a vitamin B12 and zinc dependent enzyme, methionine synthase (MTR). In alternative, mainly in the liver and in kidneys, remethylation of homocysteine to methionine can be achieved via betaine-homocysteine methyltransferase (BHMT) using the methyl group carried by betaine (trimethylglycine) produced from choline in mitochondria^11^. Methionine is converted to S-adenosyl methionine (SAM) through addition of adenosine triphosphate. SAM is the universal methyl donor and is required for methylation of DNA, RNA, histone and non-histone proteins, and lipids. SAM, after donating the methyl group to substrates, is converted into S-adenosyl homocysteine (SAH), which is hydrolyzed back to homocysteine and adenosine^12–15^. In alternative to remethylation, homocysteine can feed the de-novo biosynthesis of glutathione (GSH) by the trans-sulfuration pathway^16^ that involves two pyridoxal 5′-phosphate (PLP, vitamin B6) dependent enzymes, cystathionine β-synthase (CBS) and cystathionine γ-lyase (CSE)^17^. The balance of these reactions and the partitioning of homocysteine between re-methylation and trans-sulfuration may change according to the tissues, natural/genetic kinetic properties of the related enzymes and to the varying concentrations of the relevant substrates and co-factors. These include methylcobalamin, 5-methyltetrahydrofolate, flavin adenine dinucleotide and PLP, all belonging to the group of B-vitamins^18^.

**Figure 1:**
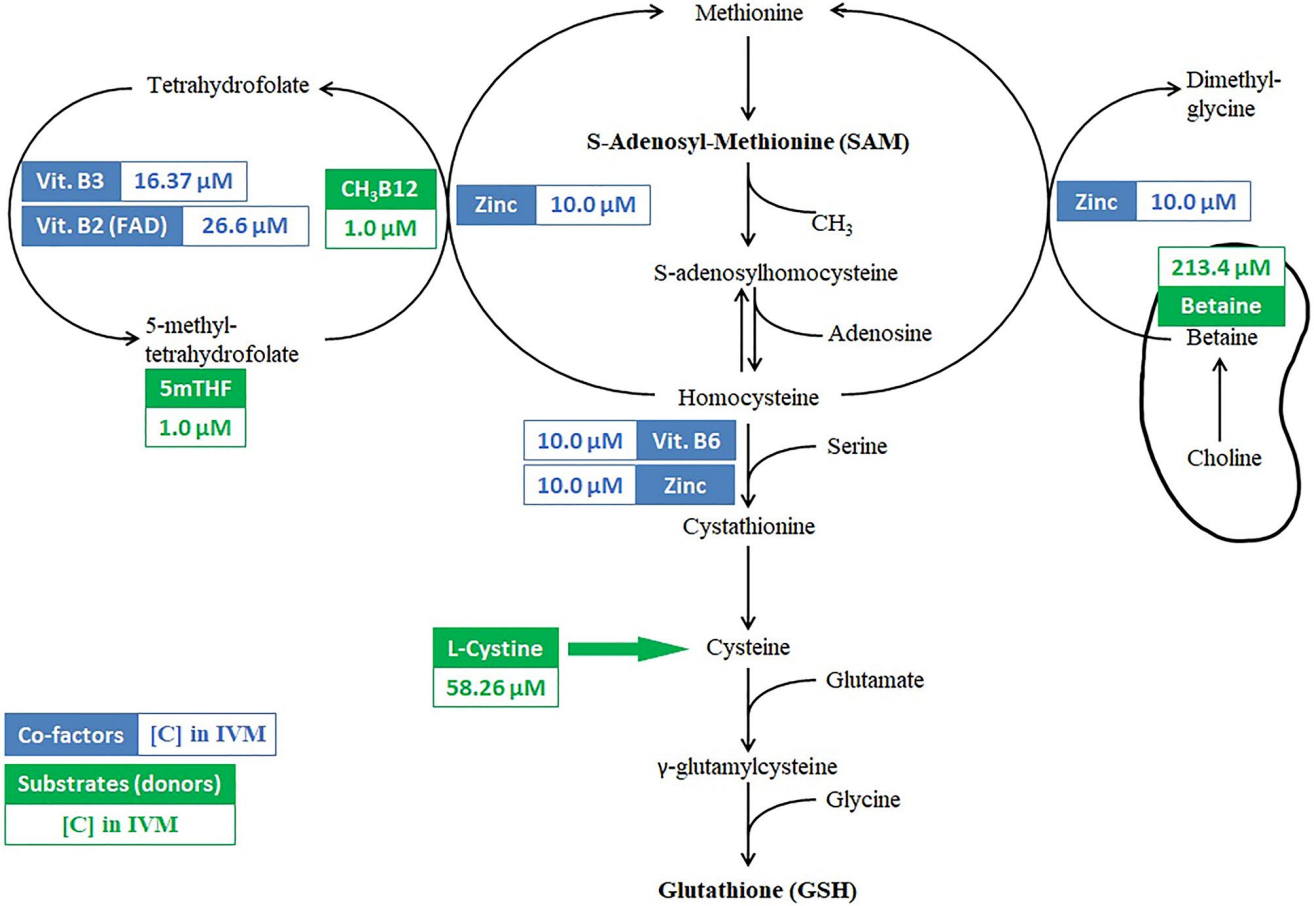
Schematic representation of the OCM pathways. The eight supplemented metabolic enhancers are reported, together with their concentrations, besides the specific reactions that they feed.

**Figure 2:**
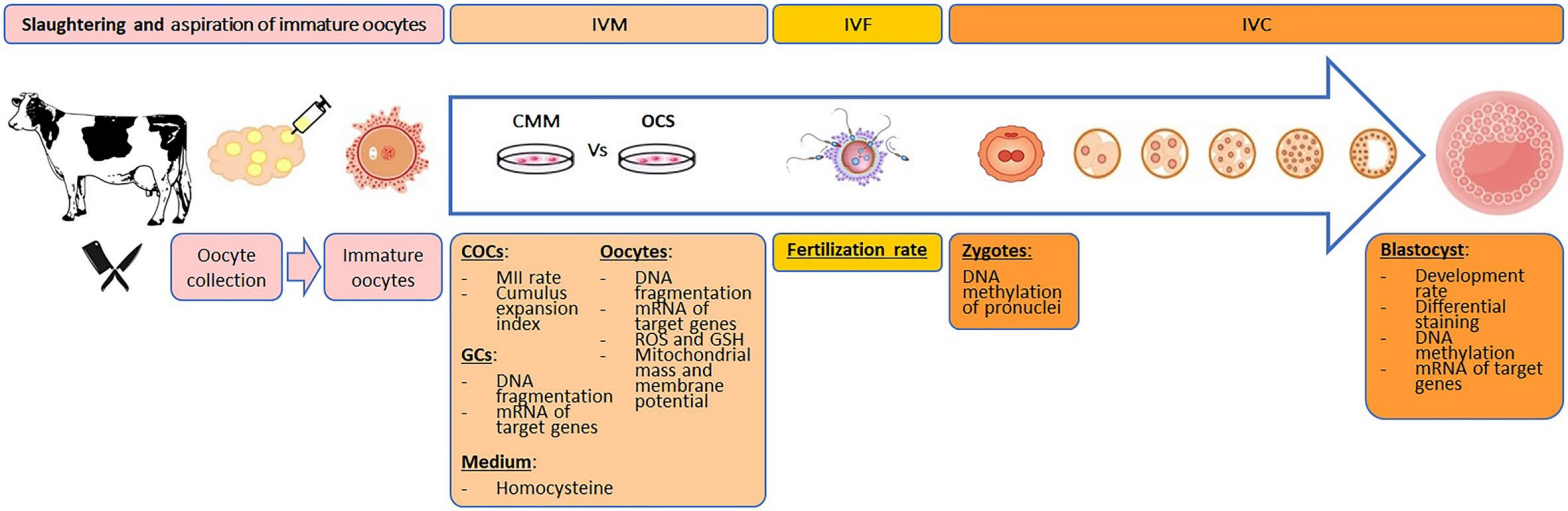
Experimental design and assessments. *CMM* Conventional maturation medium, non-supplemented (control), *OCS* The same medium supplemented with one carbon substrates and cofactors, *COCs* cumulus-oocyte complexes, *GCs* Granulosa cells, *MII* Matured oocyte, *ROS* Reactive oxygen species, *GSH* Glutathione, *IVM* In vitro maturation, *IVF* in vitro fertilization, *IVC* in vitro culture.

The importance of the up-regulation of the OCM pathways during oocyte maturation (meiosis resumption), which sustains the biosynthesis of nucleotides, proteins, and lipids and feeds the reactions of methyltransferases contributing to the epigenetic signature, prompted us to design and implement a study to detail the contribution of substrates and cofactors of the OCM to the properties of a medium for in vitro maturation (IVM). To this aim, we added to the maturation medium for bovine oocytes a set of substrates and cofactors of the OCM consisting of Betaine, Cystine, Zinc bisglycinate, Nicotinamide, Pyridoxine HCl, Riboflavin, 5-methyltetrahydrofolate (5mTHF) and Methylcobalamin (Fig. 1), to assess their effect on the pre-implantation developmental competence of the oocytes and of the resulting embryos (Fig. 2).

## Results

### Effect of OCM supplementation during the IVM of bovine oocytes on developmental competence of preimplantation IVF embryos

**Figure 3:**
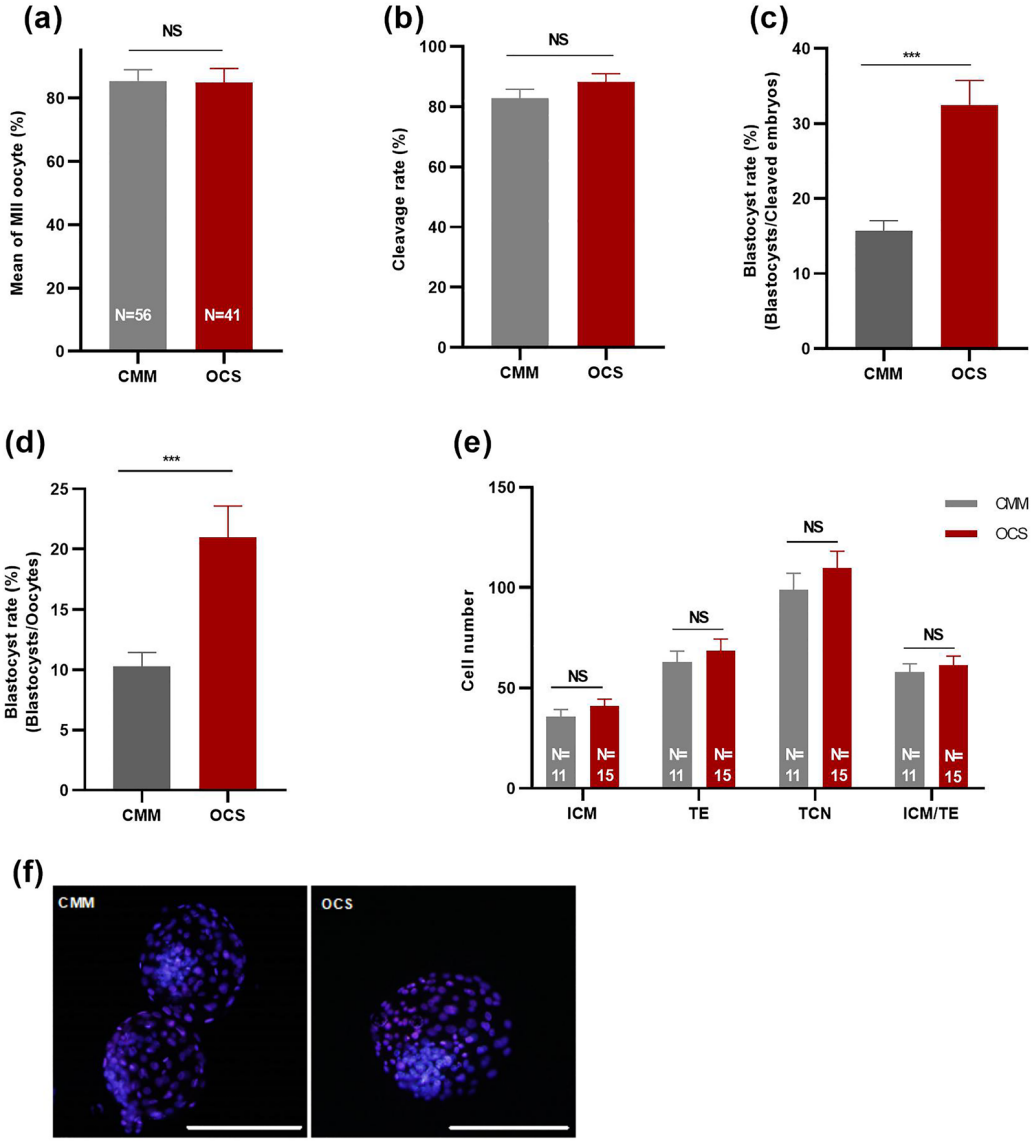
Effect of one carbon supplementation on (**a**) MII oocyte rate, (**b**) Cleavage rate, (**c**) Blastocyst rate (Blastocyst/Cleaved embryos), (**d**) Blastocyst rate (Blastocyst/Oocytes) and (**e**) Embryo cell number. (**f**) Representative images from differential staining of blastocysts of CMM and OCS groups. Magnification 20 ×; Scale bar, 100 µm. *ICM* inner cell mass, *TE* trophectoderm cells, *TCN* total cell number. The data are shown as mean ± s.e.m. NS stands for non-significant differences (*P* > 0.05). ***stands for *P* ≤ 0.001. N stands for number of total oocytes or blastocysts in each group. For embryonic development 12 replications, consisting of 700 oocytes, were done for each group.

The assessment was based on 12 replicates with 700 oocytes per group. MII rates were assessed after 24 h of IVM, cleavage and blastocyst rates were assessed at day 3 and 7 of IVC. As depicted in Fig. 3, there was no difference in MII (85.37 ± 3.56 vs 84.94 ± 4.41—Fig. 3a) and cleavage rate (82.93 ± 1.82 vs 88.26 ± 2.72—Fig. 3b) between CMM and OCS. Blastocyst rate (blastocysts/cleaved embryos) was significantly improved in the OCS (32.48 ± 3.27%) compared to CMM (15.68 ± 2.00%) group (*P* ≤ 0.001; Fig. 3c). Blastocyst rate (blastocysts/oocytes) was also significantly higher in the OCS (20.99 ± 2.61%) compared to CMM (10.28 ± 1.17%) group (*P* ≤ 0.001; Fig. 3d). The quality of blastocysts, based on total cell number and their allocation to the inner cell mass and trophectoderm, did not differ between groups (Fig. 3e,f).

### Effect of OCM supplementation on oocyte quality

**Figure 4:**
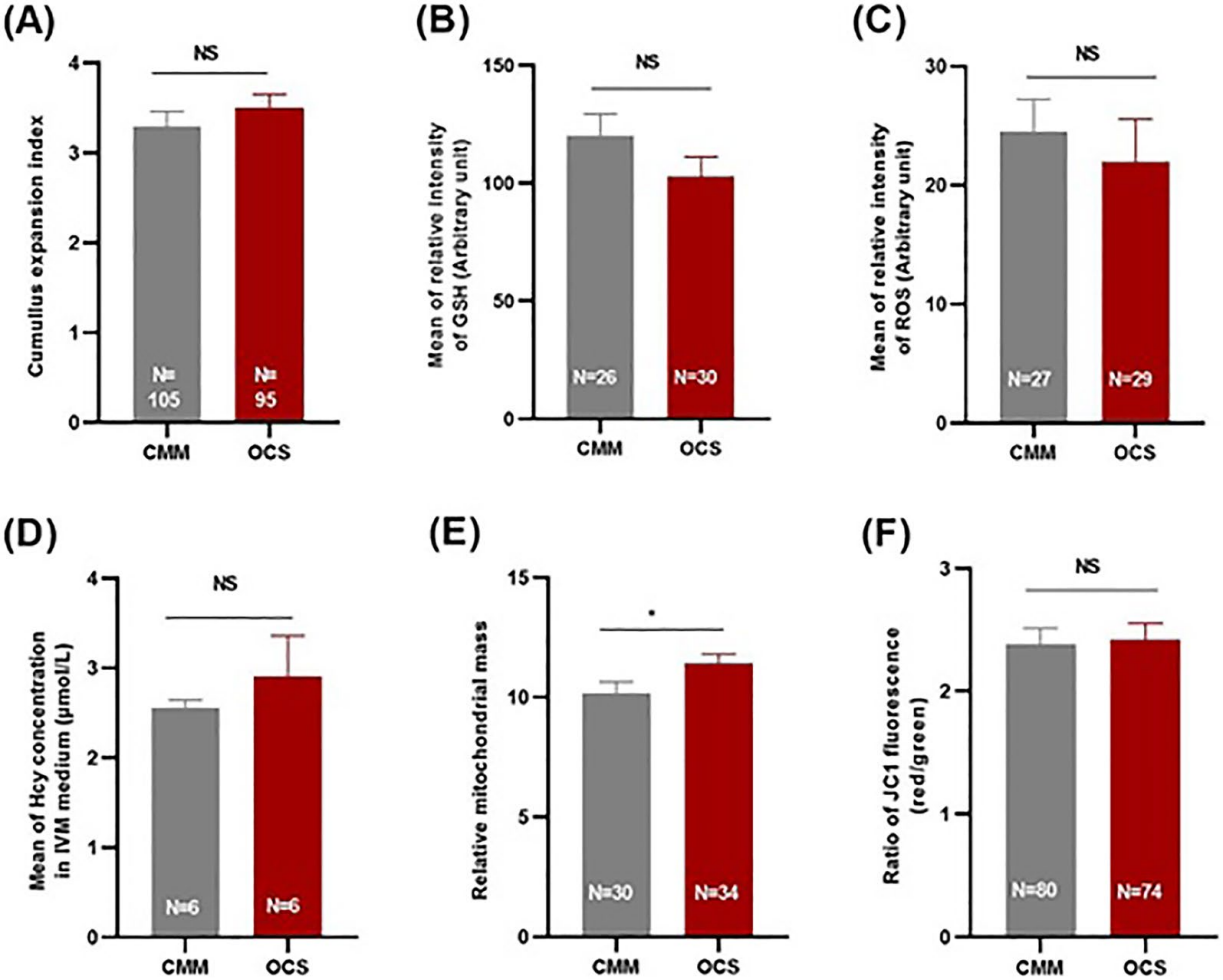
Effect of one carbon supplementation on oocyte quality. (**A**) cumulus expansion index, (**B**) relative intensity of glutathione (GSH) level, (**C**) relative intensity of reactive oxygen species (ROS) level, (**D**) homocysteine (Hcy) concentration in spent maturation medium (6 replicates), (**E**) relative mitochondrial mass and (**F**) ratio of JC1 fluorescence (red/green). The data are shown as mean ± s.e.m. NS stands for non-significant differences (*P >* 0.05). *Stands for *P < *0.05. N stands for number of total oocytes in each group (for homocysteine concentration, N stands for number of Replications).

Oocyte maturation and the completion of metaphase II are energy-demanding processes where the function of mitochondria plays a major role. In parallel, the release of ATP from mitochondria associates to a load of ROS that may cause a deficit of antioxidant defenses and damages to the nuclear DNA. Six parameters were assumed to report on the quality of the cultured oocytes, i.e. cumulus expansion index, GSH level, ROS level, homocysteine concentration, mitochondrial mass index and membrane potential (Fig. 4). The OCS group exerted a significantly higher mitochondrial mass index (11.42 ± 0.4) compared to the CMM group (10.16 ± 0.48; *P* < 0.05; Fig. 4E). The other five parameters were not significantly different between groups (Fig. 4A–D,F).

### Effect of OCM supplementation on DNA integrity of oocytes and cumulus cells

**Figure 5:**
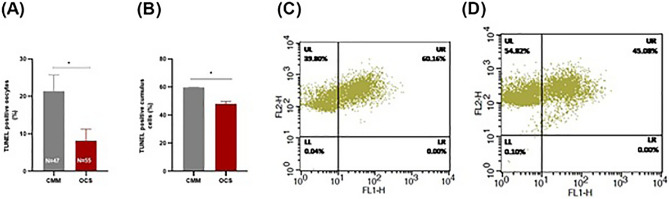
Effect of one carbon supplementation on DNA fragmentation in oocyte and cumulus cells. (**A**) TUNEL positive oocytes (%) in CMM and OCS group (4 replicates). (**B**) TUNEL positive cumulus cells (%) in CMM and OCS group (3 replicates). (**C**) Flow cytometry histograms indicating cumulus cell DNA fragmentation in CMM and (**D**) OCS. The data are shown as mean ± s.e.m. *Stands for *P* ˂ 0.05. N stands for number of TUNEL positive oocytes/total number of oocytes.

DNA integrity was assessed by TUNEL assay. Compared to the CMM group, the OCS group exerted a significantly lower number of TUNEL positive oocytes (8.15 ± 3.11% vs 21.39 ± 4.34%; *P* < 0.05; Fig. 5A) and of TUNEL positive cumulus cells (48 ± 2.00% vs 59.66 ± 0.33%; *P* < 0.05; Fig. 5B–D).

### Effect of OCM supplementation on global DNA methylation level in oocytes, in female and male pronucleus in zygotes and in blastocysts

**Figure 6:**
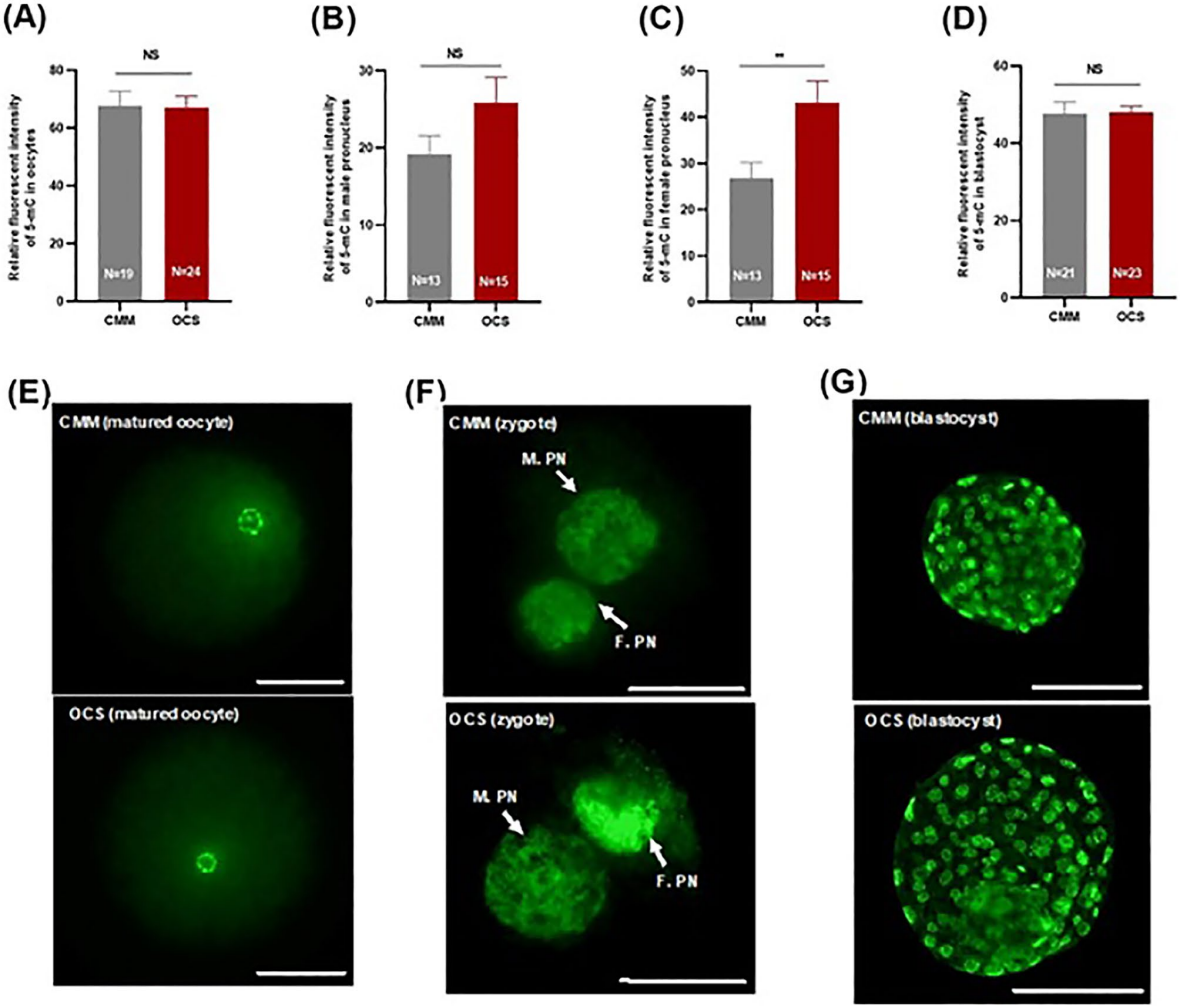
Effect of one carbon supplementation on global DNA methylation level of matured oocytes, zygotes and blastocysts. Relative fluorescent intensity of 5mC in (**A**) matured oocytes, (**B**) male and (**C**) female pronucleus of zygotes and (**D**) blastocysts. Representative images of 5-mC immunocytochemistry of (**E**) matured oocytes (40 × magnification; Scale bar, 50 µm), of (**F**) zygotes (40 × magnification; Scale bar, 50 µm) and of (**G**) blastocysts (20 × magnification; Scale bar, 100 µm). M.PN male pronucleus and F.PN female pronucleus. The data are shown as mean ± s.e.m. NS stands for non-significant differences (*P* ˃ 0.05). **Stands for *P* ≤ 0.01. N stands for total number of oocytes, zygotes or blastocysts in each group.

The acquisition of a specific epigenetic programming is a crucial part of the oocyte maturation and is largely based on DNA methylation. DNA methylation consists in the covalent binding of a methyl group donated by SAM to the 5 position of DNA’s cytosines, forming 5mC, by DNA methyltransferases (DNMTs). We assessed the DNMTs gene expression and measured the global DNA methylation level in oocytes, in female and male pronucleus in zygotes (18 hpi), and in blastocysts as the relative fluorescent intensity of 5mC. In MII oocytes there were no differences between groups (Fig. 6A,E). Unlike male pronucleus, the mean fluorescent intensity of 5mC in female pronucleus in the zygotes derived from OCS group (43.14 ± 4.65) was significantly higher than that in zygotes from the CMM group (26.8 ± 3.43; *P* ≤ 0.01; Fig. 6B,C,F). The global DNA methylation level in blastocysts derived from OCS and CMM groups was similar (Fig. 6D,G).

### Effect of OCM supplementation on mRNA expression of target genes in cumulus cells, MII oocytes and blastocysts and on DNMT3a protein expression in MII oocytes

Aiming to gain more information on the effects of the supplementation, we also investigated the transcription of key genes in the cumulus cells from matured oocytes, in matured oocytes and in blastocysts. In the cumulus cells we tested the mRNA of 3 key genes in the OCM, all involved in homocysteine re-cycling by either re-methylation, *MTR* and *BHMT*, or trans-sulfuration, *CBS*, and of 2 genes related to oocyte maturation, *HAS2* and *TNFAIP6*. In the matured oocytes we tested the expression of the same key enzymes of the OCM, *MTR*, *BHMT* and *CBS*, and the DNA methyl transferases *DNMT1* and *DNMT3a*. DNMT3a protein expression was assessed in oocytes too. In blastocysts, we also assessed cell lineage-related transcription factors involved in blastocyst formation, *POU5F1* and *NANOG* as pluripotency markers and *TEAD4* as a trophectodermal marker.

**Figure 7:**
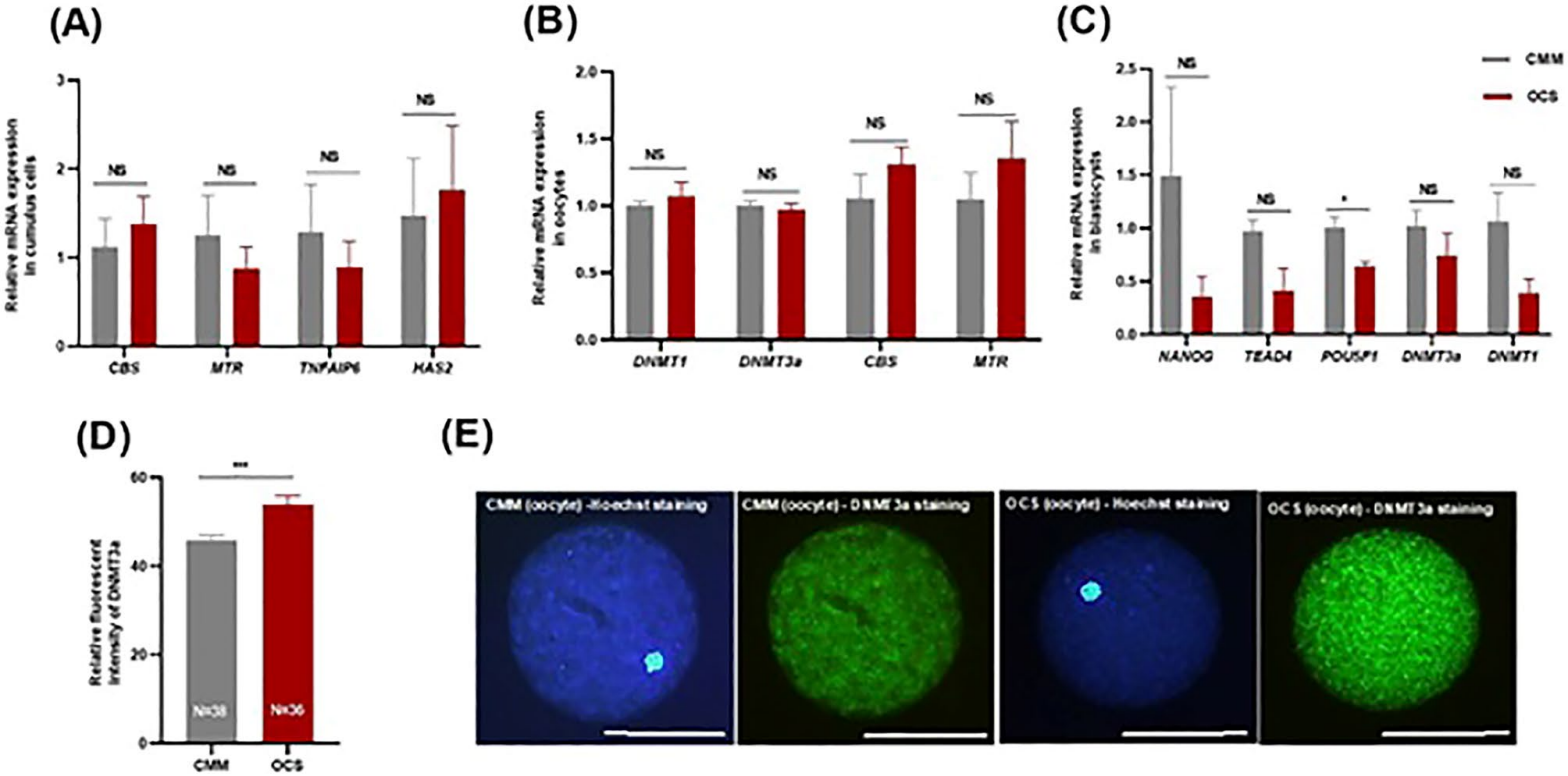
Effect of one carbon supplementation on relative mRNA expression of target genes in (**A**) cumulus cells, (**B**) matured oocytes and (**C**) blastocysts. (**D**) Effect of one carbon supplementation on DNMT3a protein expression in terms of fluorescent intensity in matured oocytes. (**E**) Representative images of DNMT3a immunocytochemistry in matured oocytes (20 × magnification; Scale bar, 100 µm). The data are shown as mean ± s.e.m. NS stands for non-significant differences (*P > *0.05). *Stands for *P* < 0.05. ***Stands for *P* ≤ 0.001. N stands for total number of oocytes in each group.

There was no difference between groups in target genes assessed in the cumulus cells (*CBS, MTR*, *BHMT, TNFAIP6* and *HAS2*) and in oocytes (DNMTs, *CBS, MTR* and *BHMT)* (Fig. 7A,B). Among the target genes assessed in blastocysts (*NANOG, TEAD4, POU5F1* and DNMT*s*), only *POU5F1* showed significantly lower expression in OCS compared to CMM group (*P˂0.05*; Fig. 7C). The expression of the DNMT3a protein in MII oocytes was significantly higher in OCS (53.90 ± 1.98) compared to CMM (45.84 ± 1.11: *P* ≤ 0.001; Fig. 7D,E).

## Discussion

The sensitivity of oocyte IVM to the function of the OCM was already reported in the literature. Li et al. found that some important metabolites of the OCM pathways including S-adenosylmethionine (SAM), 5′-methylthioadenosine (MTA) (in the methionine cycle), glutamate and pyroglutamate (in the trans-sulfuration pathway) were upgraded in mice oocytes during in vivo maturation together with an increased expression of enzymes related to the folate cycle, the methionine cycle, and the trans-sulfuration pathway^8^. Their findings demonstrated the increased demand for OCM activity during oocyte maturation raising the question of whether the same metabolism should be duly supported also in case of maturation in vitro.

Previous attempts to provide such a support had focused on the supplementation of single metabolic enhancers^19,20^ and resulted in increased blastocyst rate. Our support appeared more effective as resulting in a 107% increase of the blastocyst rate, which may depend on the use of multiple enhancers and/or on a putative higher sensitivity of the bovine species to folates. However, another relevant difference applies: Both the studies supplemented folates as FA whereas we used, for the first time, the natural, soluble and circulating substance: 5mTHF^9^.

FA is an industrial surrogate^21^ that does not naturally occur in mammalian metabolism. In humans, it is slowly converted to dihydrofolate with the potential to behave as a pathway inhibitor at clinically achievable concentrations^22^. Nowadays, despite the confirmed importance of prenatal and postnatal folate intake^23–25^, growing concerns about high and non-natural unmodified FA (UMFA) blood concentrations in populations exposed to food fortification with FA have been expressed^26–31^. High blood level of UMFA can cause metabolic imbalances that may damage embryogenesis: High dietary FA in pregnant mice led to pseudo-MTHFR deficiency and altered methyl metabolism, which subsequently delayed embryonic growth and impaired the short-term memory in offspring^32^. In addition, FA can induce an excess of folate intermediate metabolites that may undergo oxidation to release formaldehyde, which triggers DNA de-methylation^33^ and may hamper embryo epigenetics. Thus, the substitution of FA with 5mTHF is among the possible reasons for an increased efficiency of our support. Accordingly, future studies on in vitro oocyte and embryo support should now focus on the use of 5mTHF, even more if attempting at the human translation of the data.

Previous studies have shown that the function of the OCM is essential for a proper epigenetic programming of stem cells^34^. Thus, we hypothesized that enhancing the OCM could modify the number of ICM and TE and also their ratio. However, despite the positive effect of one carbon supplementation on the blastocyst rate, the quality of our blastocyst in terms of number of ICM and TE and of ICM:TE ratio did not differ between CMM and OCS groups. This might depend on the shortness and timing of our OCM supplementation, only 24 h during oocyte maturation, with the epigenetic programming leading to the ICM and TE differentiation occurring thereafter in zygotes that were not anymore supplemented, i.e. our treatment may have been enough to trigger the development but too short to induce measurable differences in the blastocyst cell mass and distribution. However, an alternative explanation is possible: A OCM efficiency checkpoint is in place to select zygotes that can efficiently further develop, i.e., only those oocytes that were able to run the OCM with the available amounts of substrates during IVM will be able, once fertilized, to properly activate the embryonic genome. Indeed, the actual efficiency in using the OCM substrates is genetically determined by the specific variants of the OCM enzymes^9^ so that only the oocytes/zygotes with the best suited genetic inheritance would properly work with the limited amounts of substrates available in vitro and would pass the checkpoint. Obviously, a wide supplementation of the OCM substrates may decrease the threshold for OCM efficiency allowing a larger number of zygotes to progress. Thereafter, given that only winning configurations passed the checkpoint, no quality difference would be visible anymore once the blastocyst state is reached. In summary, a OCM checkpoint for pre-implantation embryos seems to be in place and our supplementation might have simply decreased the proportion of COCs that are lost during IVM and the following early stages of pre-implantation development. Accordingly, a longer support, beyond the 24 h of in vitro maturation of our study, could further improve the yield of embryo IVP and should be properly investigated.

In cumulus cells and in matured oocytes there were no differences in the expression of the tested genes and *BHMT* was not at all expressed. In mouse pre-implantation embryos, *BHMT* mRNA was well expressed from morula up to the blastocyst stage with a peak of BHMT protein expression in the blastocyst^35^. We tested *BHMT* expression only in cumulus cells and in matured oocytes, i.e. out of the expression window described in mice, hence we cannot confirm a similar expression profile in bovines. However, it remains possible and likely that our betaine supplementation during IVM may have contributed to the accumulation of betaine in the oocytes and to the following methylation job at time of blastocyst expansion.

In the blastocysts we tested the expression of *DNMT1* and *DNMT3a*, which did not show any differences between groups, likely because, due to the OCM check point, only blastocysts with a proper expression of *DNMT*s had developed. We also tested cell lineage-related transcription factors involved in blastocyst formation. Ikeda et al. showed that the supplementation of a bovine embryo culture medium with ethionine, an antimetabolite of methionine, did not affect *POU5F1* and *CDX2* expression but increased the expression level of *NANOG* and *TEAD4* that disturb TE/ICM lineage segregation and prevent cell differentiation^36^. We found lower expression of *POU5F1* in OCS as compared to CMM group blastocysts. The expression of *POU5F1* is known to be progressively repressed in developing blastocysts, to trigger cell differentiation, by processes of targeted methylation of the gene promoter^37^. Therefore, it is possible that the improved methylation potential in our OCS group blastocysts may have favored a better-timed trigger of the cell differentiation processes as marked by the reduced expression of POU5F1.

Oocytes from the supplemented group (OCS) exerted an increased mitochondrial mass without any changes of the membrane potential. The mitochondria of oocytes are at rest with little bioenergetic going on up to fertilization, which is believed to preserve mitochondrial DNA from oxidative damages: They are only required to grow in number so to distribute to all blastomeres after fertilization^38,39^. Thus, the increased mitochondrial mass without any premature changes of the MMP resulting from OCS is well fitting with and possibly playing a role in the increased ability to expand to blastocyst later on.

Cysteine is the substrate for GSH synthesis and is achieved both from the diet and from trans-sulfuration of homocysteine by the enzymes CBS/CSE. We supplemented both cysteine (as cystine) and the CBS cofactors vitamin B6 and Zinc to further aid homocysteine conversion to cysteine. Moreover, the supplementation of methyl donors (5mTHF, methylcobalamin, betaine) should have favored the release of an excess of SAM for a full activation of CBS and of GSH de-novo synthesis^9^. Nevertheless, the GSH level did not show any difference after OCM supplementation. In contrast, in another study, the supplementation of porcine oocyte maturation medium with 10 ng/ml FA significantly increased GSH content of matured oocytes^19^. However, the oocytes had been cultured with FA for 44 h whereas we tested GSH after only 24 h of exposure to the metabolic enhancers. Hence, the activation of GSH release may simply need longer time to be measurable. Moreover, we could not account for a possible increased release of GSH from supplemented cumulus cells, which may have helped in reducing the DNA fragmentation in the oocytes. Finally, GSH migrates to mitochondria at time of energy demand and ROS release and to the nucleus during mitosis and meiosis^40^. We dosed GSH by Cell tracker Blue that may fail to precisely report on GSH concentration if extreme compartmentalization is in place, therefore it remains possible that enhanced GSH availability in mitochondria and nuclei, although not resulting in a concentration difference by Cell Tracker Blue staining, was responsible for the decreased DNA damage that we recorded in supplemented oocytes. As a matter of fact, the supplementation of oocytes IVM with metabolic enhancers of the OCM strongly decreased the DNA damage to the oocytes. This effect may have contributed to the higher blastocyst rate of the same oocytes and is likely to extend its benefit also beyond the pre-implantation stage, i.e. in pregnancy survival, which could not be assessed in our model.

Any imbalance of the OCM is known to result in homocysteine accumulation and this may have a strong negative role on the reproductive function. Moreover, homocysteine and methionine compete for the same amino acid transporter so, high homocysteine will mean low methionine uptake^41^, further hampering the OCM. Berker et al. found that no pregnancies occurred in those patients whose follicular fluid homocysteine was above 8 µM^42^. In our experiments, homocysteine in spent IVM medium did not differ between our OCS vs CMM oocytes, which appears to negate the induction of the OCM. However, homocysteine concentration in our spent media was 2–3 µM, i.e. well below the amount accumulated during the whole pre-ovulatory period in the follicular fluid of either pregnant or non-pregnant patients in the mentioned study. Thus, our lower homocysteine level may simply mean that our culture time period of 24 h was not long enough to induce a measurable homocysteine accumulation.

In oocytes, *DNMT3a* is used for de novo methylation and *DNMT*1 is responsible for DNA methylation maintenance. We found no difference between the mRNA expression level of *DNMT1* and *DNMT3a* between the CMM and the OCS groups. However, at this stage the process was supposed to be already completed and, according to the OCM checkpoint concept, only oocytes achieving a sufficient epigenetic machinery were expected to mature and to expand to blastocyst. Moreover, the transcriptome is an indirect reporter with many possible post-transcriptional modifications influencing the actual phenotype. Indeed, we found that the DNMT3a protein was more abundant in the oocytes matured with the support of the OCM enhancers and this may have favored an improved DNA methylation profile.

When we looked at the 5mC amount, i.e. DNA methylation level, of our in vitro matured oocytes and in their blastocysts, we found no differences between supplemented and non-supplemented IVM. However, differently from Duan et al. using bisulfite sequencing^43^, we detected 5mC by antibodies that may have not properly penetrated the tightly packed chromatin of MII oocytes. Indeed, when we looked at the methylation level of the male and female pronuclei in the zygotes, i.e. after the initial chromatin de-condensation that follows the fertilization, our antibodies were able to demonstrate an increased amount of 5mC in female pronuclei. This finding directly endorses the feeding role of our metabolic enhancers during IVM and fits with the increased expression of the DNMT3a protein. In summary, the support of the IVM medium with metabolic enhancers of the OCM was able to increase the DNA methylation of oocytes, although this difference did not play a role in their ability to reach the MII stage, which was similar between the groups.

The above difference in DNA methylation status disappeared when we analyzed the blastocysts resulting from the same differently methylated groups (CMM vs OCM) of MII oocytes, which appears as an evident effect of the OCM checkpoint. Our IVM medium supplementation was indeed of benefit to the oocytes only during the 24 h of IVM and could not deliver a direct feeding to the zygote after fertilization, i.e. any benefit was a carry-over effect from the initial priming during IVM. Such priming did not appear to be primarily involving the redox balance, as we found no differences between groups in GSH, ROS and homocysteine, but appeared to involve the methylation dynamics as marked by increased expression of the DNMT3a protein and increased DNA methylation in female pronuclei of zygotes. Such priming, which did not affect the MII rate of oocytes neither the embryo cleavage rate that both occur before the activation of the embryonic genome, played a strong effect in the following ability to progress to the blastocyst stage that increased by 107% with no quality difference among the developed blastocysts.

**Figure 8:**
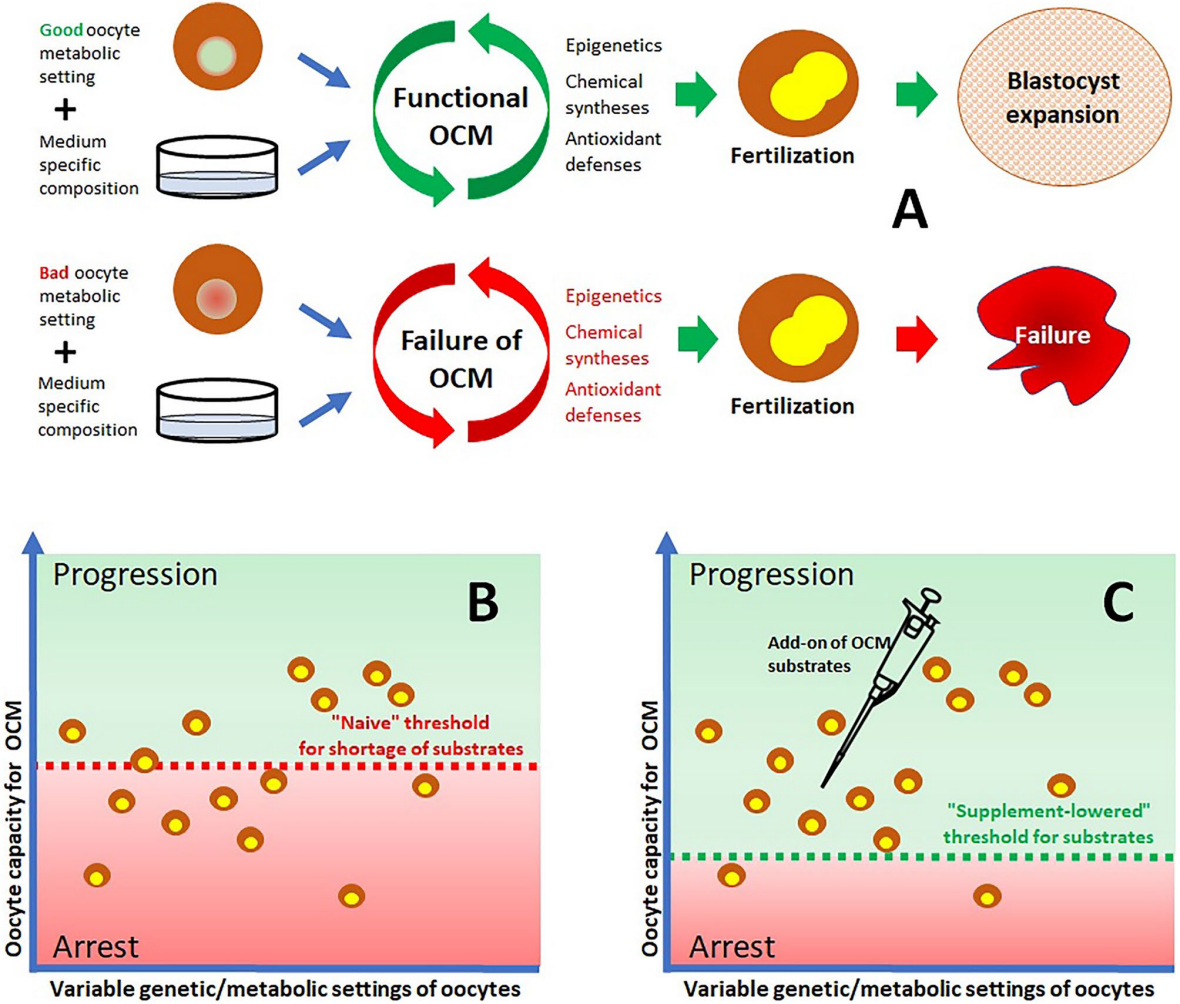
The one carbon metabolism (OCM) checkpoint. (**A**) Each maturing oocyte has its own, genetically determined, capacity to process the OCM substrates. In in vitro conditions the variety and amounts of substrates available to run the OCM is the one provided by the culture medium, and it may not fit with the need of some oocytes. Oocytes well-fitting with their medium will achieve maturation, fertilization and, thereafter, blastocyst expansion. Oocytes with a bad fit with the medium will suffer a deficit of function of the OCM and, although able to mature and to get fertilized, will fail the full development to blastocyst. (**B**) Out of a cohort of oocytes undergoing IVC, some of them will have good ability to process OCM substrates, other will need larger amounts. The specific threshold of different oocytes is reported by their position with respect to the vertical axis. Any oocyte positioned above the available concentration of substrates (dotted line, green background area) will be able to run the OCM and to progress. All those below the dotted line (red background area) are predicted to fail to expand to blastocyst after fertilization. (**C**) The maturing environment (culture medium or in vivo) of the same cohort of oocytes has been supplemented with due amounts of the OCM substrates. Even if the intrinsic metabolic capacity of the oocytes remains the same, the supplementation of OCM substrates lifts-up their concentration, which allows more of oocytes to fall within the progression area and to expand to blastocyst once fertilized.

Based on the gamete’s specific genetic inheritance of functional variants of the enzymes of the OCM, oocytes and zygotes (with the contribution of sperm genetics) have different minimum requirements for the OCM substrates: Only those oocytes/zygotes whose minimum requirement is satisfied by the nutrient feed from the follicular/tubal fluid in vivo or from the maturation medium in vitro will be able to pass the checkpoint and to move for blastocyst expansion. Feeding the IVM medium with extra amounts of the same substrates was able to lower the threshold for development so to allow a larger number of zygotes to progress, which accounts for the increased blastocyst rate that we recorded. The concept of the OCM checkpoint is depicted in Fig. 8. The variable assembly of genetic variants of the OCM genes/enzymes occurring in humans^9^ is likely to result in a similarly variable metabolic suitability of human gametes and blastocysts and supports to the OCM metabolism have the potential to be of benefit also in the clinical setting.

## Conclusion

In this study, we supplemented oocyte maturation medium with 8 substrates and co-factors for enhancing the one carbon metabolism to optimize the culture system. Although no difference in oocyte maturation rate was recorded, this supplementation resulted in lower DNA fragmentation and higher mitochondrial mass and DNMT3a protein expression in oocytes and in improved DNA methylation in female pronucleus of zygotes, which associated to an improved rate of development to blastocyst. The strongly increased blastocyst rate in front of no difference in MII rate strongly points to the existence of a OCM metabolic checkpoint that works already during oocyte final maturation to exert its selective effects thereafter, i.e. by limiting the number of mature oocytes that are able to progress after fertilization. A proper support of the oocyte maturation medium with metabolic enhancers may help to overcome the OCM checkpoint barrier and to improve the yield from oocyte IVM. Similar benefits might occur supplementing also the embryo culture in vitro and the gametogenesis/embryogenesis in vivo, which warrants further investigations.

## Materials and methods

### Ethics

All animal care protocols and all the experimental protocols used in this study (Proposal No. 99000086) were handled according to the guidelines and regulations provided by the Institutional Review Board and Institutional Ethical Committee of the Royan Institute. Also, the manuscript follows the recommendations in the ARRIVE guidelines. In addition, we clarify that no experiments on humans and/or the use of human tissue samples involved in this study.

### Media and reagents

All chemicals and media were obtained from Sigma Aldrich Chemical Co. (St. Louis, MO) or Gibco (Grand Island, NY), unless otherwise stated.

### Experimental design and endpoint analyses

Bovine cumulus oocyte complexes (COCs) were aspirated from ovaries collected from abattoir and matured in vitro in either a conventional maturation medium (CMM) or in a maturation medium supplemented with eight substrates and cofactors of one carbon cycle (one carbon metabolism supplemented; OCS). Matured oocytes were then fertilized in vitro and cultivated up to blastocyst development. In the OCS group the medium was supplemented with Betaine (213.4 µM^44^), Cystine (58.26 µM^45^), Zinc bisglycinate (10 µM), Nicotinamide (16.37 µM^46^), Pyridoxine HCl (10 µM), Riboflavin (26.6 µM), 5-methyltetrahydrofolate (5mTHF) (1 µM) and Methyl cobalamin (1 µM) (Please see Fig. 1).

After 24 h of in vitro maturation, the MII rate and cumulus expansion index were assessed in matured COCs. MII oocytes were tested for reactive oxygen species (ROS), glutathione (GSH), mitochondrial mass and membrane potential, DNA methylation rate and relative expression of DNMT3a protein. DNA fragmentation rate and mRNA expression of target genes were evaluated in both matured oocytes and in cumulus cells. Homocysteine concentration in spent maturation medium was also assessed.

The achieved zygotes were assessed for the global DNA methylation rate and cultivated up to the blastocyst stage. The blastocyst rate was calculated and blastocysts were investigated by differential staining, global DNA methylation and mRNA expression of target genes. The experimental design and procedures are depicted in Fig. 2.

### In vitro oocyte maturation

Bovine cumulus oocyte complexes (COCs) were aspirated from 2 to 8 mm follicles of slaughterhouse ovaries through an 18-gauge needle attached to a vacuum pump (80 mm Hg). The aspiration medium was the HEPES-buffered TCM-199 supplemented with 10% fetal bovine serum (FBS) and heparin (10 μl/ml). COCs with homogenous cytoplasm, intact zona pellucida and cytoplasmic membrane and at least 3 layers of compact cumulus cells were selected and randomly assigned to maturation in either CMM or OCS group.

Selected COCs were incubated for 24 h in a maturation medium consisting of TCM supplemented with 10% FBS, 10 µg/ml follicle-stimulating hormone (FSH), 10 µg/ml luteinizing hormone (LH), 2.5 mM sodium pyruvate, 1 µg/ml estradiol-17β, 10 ng/ml epidermal growth factor, 0.1 mM cysteamine and 10 μg/ml insulin-like growth factor (IGF, R&D, USA) at 38.5 °C, 6% CO_2_, and maximum humidity. In treatment group (OCS), conventional oocyte maturation medium was also supplemented with eight substrates and cofactors of the OCM with defined concentration as detailed earlier in the experimental design.

### In vitro fertilization (IVF) and in vitro culture (IVC)

Following maturation, the IVF procedure was performed as described previously^47^. Briefly, motile sperms were prepared using swim down method with PureSperm gradient. One million /ml sperms were co-incubated with 10 matured COCs in 50 μl fertilization medium for 18–20 h at 38.5 °C, 5% CO_2_ and humidified atmosphere.

After 18–20 h post insemination, COCs were mechanically denuded of cumulus cells and cultured in synthetic oviduct fluid (SOF) supplemented with ITS (insulin, transferrin, selenium) and myo-inositol (modified SOF: mSOF). The resulting zygotes underwent IVC in mSOF without glucose and serum (mSOF^−^) for 3 days at 38.5 °C, 6% CO_2_, 5% O_2_ in humidified air under mineral oil. On Day 3, the cleavage rate was assessed. Thereafter, the embryos were transferred to SOF medium supplemented with glucose and serum (mSOF^+^) and the blastocyst rate was evaluated on Day 7 and 8*.* The rate of blastocyst improvement was calculated as: (blastocyst rate in treatment group − blastocyst rate in control group)/blastocyst rate in control group.

### Cumulus expansion index

Twenty-four hours post IVM, cumulus expansion index was scored on a scale ranging 0 to 4 as described previously^48^: Score 0, no expansion observed in cumulus cells; Score 1, no expansion in cumulus cells but with appearance of spherical cells; Score 2, expansion only the outer layers of CCs; Score 3, expansion of all layers of cumulus cells except the corona radiata, and; Score 4, expansion of all layers including corona radiata.

### Evaluation of oocytes arrested at MII stage

In order to assess the maturation rate following IVM, cumulus cells were removed from COCs by vortexing with 300 IU/ml hyaluronidase. The denuded oocytes were washed 3 times in phosphate buffer solution without calcium and magnesium (PBS-B) containing 1 mg/ml polyvinyl alcohol (PBS/PVA), afterward fixed for 20 min in 4% paraformaldehyde. Then, they were stained with Hoechst 33342 (10 µg/ml) for 5 min. After mounting the stained oocytes on glass slide, the image of the stained oocyte was captured and assessed by a high-resolution digital camera (DP-71 Olympus) using DP2‐BSW software (provided by the instrument manufacturer).

### Measurement of relative ROS and GSH levels

GSH and ROS levels of oocytes were measured as previously described^49,50^. Following COCs maturation, matured COCs were first denuded and then zona pellucida was removed from oocyte using proteinase solution. Zona free oocytes were exposed to 2.5 mM 2, 7‐dichloro dihydroflourescein diacetate (H2DCFA) for 1 h, to detect ROS, or 10 µM Cell Tracker Blue CMF2HC for 30 min, to detect GSH, at 38.5 °C in the dark. After washing in PBS/PVA, stained oocytes were placed into 10‐µl drops of PBS/PVA and observed by an inverted fluorescent microscope (IX71; Olympus, Tokyo, Japan). A digital image of each oocyte was taken through a highly sensitive camera (DP‐72; Olympus) using DP2‐BSW software. The fluorescence intensity was analyzed through Image J software (National Institutes of Health, Bethesda, MD; https://imagej.nih.gov/ij/). ROS and GSH assessments were performed in triplicates.

### Relative mitochondrial mass

Mitochondrial mass was evaluated by Mitotracker green (MTG, Invitrogen/Molecular Probes, Eugene, OR) that integrates into the mitochondrial lipid compartment regardless of the membrane potential, reporting on the whole mitochondrial mass^51,52^. Briefly, zona free oocytes were kept in PBS/PVA containing 500 nM MTG for 1 h at 38.5 °C. Then, oocytes were washed several times in PBS/PVA medium and placed individually in drops of PBS/PVA. The fluorescence intensity of MTG (Excitation/Emission: 490/516 nm) was determined for each oocyte individually using an inverted fluorescence microscopy (Olympus BX51, Tokyo, Japan). A digital image of each oocyte was taken through a highly sensitive camera (DP‐71 Olympus). The Fluorescence intensity of MTG was measured using Image J software. The mitochondrial mass measurement was performed in triplicates.

### Mitochondrial membrane potential

Mitochondrial membrane potential (MMP) was assessed using JC‐1 (JC‐1 fluorochrome; Cat. No. M34152; Invitrogen, Carlsbad, CA) according to the manufacturer’s protocol. Briefly, zona free oocytes were transferred to HTCM medium with JC1 (20 µg/ml) and cultured for 1 h in a 5% CO_2_ incubator at 38.5 °C. After 1 h, oocytes were washed several times in PBS/PVA medium. Then, oocytes were placed in drops of PBS/PVA individually and fluorescence intensity of JC1 aggregates and monomer was determined for each oocyte individually using an inverted fluorescence microscopy (Olympus BX51, Tokyo, Japan). A digital image of each oocyte was taken through a highly sensitive camera (DP‐71 Olympus). Fluorescence intensities of aggregates and monomer were measured using Image J software. The ratio of aggregates and monomer was calculated as the average intensity of aggregates divided by the average intensity of monomer. The JC1 measurement was performed in triplicates.

### Detection of DNA-fragmentation in oocytes and cumulus cells by TUNEL assay

In order to detect DNA fragmentation in oocytes, TdT-mediated dUTP-digoxigenin nick end labeling (TUNEL) assay was carried out (in Situ Cell Death Detection Kit, Promega Corporation, USA) according to the manufacturer’s protocol with some modifications. In brief, after dissociation of cumulus cells and washing in PBS/PVA, oocytes were fixed in 4.0% paraformaldehyde at room temperature (RT) for 20 min. then, the fixed oocytes were washed three times with PBS/PVA and permeabilized in 0.5% (v/v in PBS-B) Triton X-100 for 30 min at room temperature. Then, after equilibration in buffer for 10 min at room temperature, they were placed in TUNEL mix (equilibration buffer, nucleotide mix, and rTdT enzyme) at 38.5 °C for 1 h in the dark. In order to stop the reaction, oocytes were transferred into 2X buffer for 15 min. Then, oocytes were incubated in 10 μg/ml RNase A for 1 h at room temperature in the dark. At the end, the nuclei of oocytes were stained with 1 μg/ml propidium iodide. The oocytes were then washed in PBS/PVA, mounted with coverslips and examined under a fluorescence microscope (Olympus BX51, Tokyo, Japan). The ratio of TUNEL labeled oocytes to total examined oocytes was calculated as DNA fragmentation index (DFI).

The detection of DNA fragmentation by TUNEL assay in cumulus cells was similar to the one in oocytes. Initially, 6 × 10^6^ cumulus cells from 300 matured oocytes (in each replicate) in both CMM and OCS groups were fixed with 4.0% paraformaldehyde and permeabilized with Triton-X-100. After that, cumulus cells were incubated with TUNEL mix in humidified box for 1 h at 38.5 °C in dark. After stopping the reaction with 20X SSC (a buffer consisting of 87.7 g NaCl and 44.1 g sodium citrate dissolve in 400 ml of deionized water), the cumulus cells were treated with RNase A. Finally, cells were filtered through 40 µm nylon mesh in order to exclude aggregation. Ten thousand cells were collected with the FACS-Caliber and were analyzed using CELL QUEST 3.1 software (Becton Dickinson) (provided by the instrument manufacturer). Three replicates were conducted for each treatment with appropriate controls to eliminate the possible effects of autofluorescence.

### Evaluation of cumulus mRNA expression

The expression of genes related to oocyte maturation, hyaluronon synthase 2 (*HAS2*) and tumor necrosis factor alpha-induced protein 6 (*TNFAIP6*), and genes involved in regulation of the OCM, *CBS*, *BHMT* and* MTR*, were analyzed using real time reverse transcription polymerase chain reaction (RT-PCR).

Total RNA of cumulus cells from 30 matured COCs was extracted using RNeasy Mini Kit (QIAGEN, Germany, 74104) for each replicate according to the manufacturer’s protocol. Three independent biological repetitions were carried out. Total RNA was reverse transcribed using a cDNA Synthesis kit (Yektatajhiz, Iran, YT4500) according to the manufacturer’s protocol. Quality and integrity of cDNA was checked using PCR and housekeeping primer (*B-ACTIN*), as a reference gene in the RT-PCR analyses. Three technical replicates were performed for each sample and the mean cycle threshold (Ct) was calculated. Relative expression was computed using Ct values that were normalized against *B-ACTIN*. Fold change in gene expression was calculated using 2^−ΔΔCT^. All the primers were designed by Primer 3 program (http://primer3.ut.ee/) and their characteristics are listed in supplementary information (Table S1).

### Evaluation of mRNA expression in oocytes and embryos

The mRNA expression of DNA methyltransferases genes *DNMT1* and *DNMT3a* was analyzed in both oocytes and embryos. In oocytes, also the expression of genes involved in regulation of one carbon metabolism, *CBS, BHMT* and *MTR*, was analyzed. Furthermore, to assess the quality of the resulting embryos, the mRNA expression of *POU5F1* and *NANOG* (pluripotency markers) and *TEAD4* (trophectodermal marker) were analyzed in blastocysts.

To prepare the pools of matured oocytes, at the end of oocyte maturation, COCs were harvested and cumulus cells were dissected with 300 IU/ml of hyaluronidase. After washing the denuded oocytes with PBS-B, the zona pellucida was removed using proteinase solution. After that zona removed oocytes were suspended in groups of 30 in lysis buffer and stored at -80ºC. The oocytes were collected in four independent biological replications.

Blastocysts were produced by IVF in three replicates. At day 7 Blastocyst were collected, processed similarly to oocytes and suspended in groups of 6 in lysis buffer and stored at − 80 °C.

Total RNA of oocytes and blastocysts was extracted using RNeasy Micro Kit (QIAGEN, Germany, 74004) for each replicate according to the manufacturer’s protocol. Total RNA was reverse transcribed using a cDNA Synthesis kit (Yektatajhiz, Iran, YT4500) according to the manufacturer’s protocol. Quality and integrity of cDNA was checked using PCR and the housekeeping primer (*B-ACTIN*) as a reference gene. Three technical replicates were performed for each sample and the mean cycle threshold (CT) calculated. Relative expression was computed using Ct values which was normalized against *B-ACTIN*. Fold change in gene expression was calculated using 2^−ΔΔCT^. All the primers were designed by Primer 3 program (http://primer3.ut.ee/) and their characteristics are listed in Supplementary Information, Table S1.

### Homocysteine assessment in maturation medium

For homocysteine assessment, 300 µl of spent maturation medium were collected after 24 h of IVM in six independent replications. The spent maturation medium was centrifuged at 400×*g* for 5 min to exclude debris and cumulus cells. Homocysteine level was determined using chemiluminescence.

### Immunofluorescence staining for detection of DNMT3a in matured oocytes

Zona free oocytes were washed three times in PBS/PVA, fixed in 4% PFA for 20 min and washed in PBS/PVA three times. Oocytes were incubated in permeabilization solution (PBS/PVA and 1% Triton-X-100) for 30 min at RT, followed by incubation for 1 h in blocking solution: PBS/PVA containing 0.1% Triton-X-100, 1% bovine serum albumin (BSA) + 10% goat serum. Oocytes were then incubated overnight at 4 °C with DNMT3a primary antibody (DNMT3a, 64B1446, NB120-13888) in antibody solution (PBS/PVA containing 0.1% Triton-X-100 and 1% BSA). For negative control, primary antibody was replaced with the same concentration of mouse IgG. After washing extensively, oocytes were incubated with goat anti-mouse IgG conjugated with FITC (Millipore; AP124F) for 45 min at 37 °C in the dark. After each step, oocytes were washed with washing solution (PBS/PVA and 0.1% Triton-X-100). Nuclear labeling was achieved by incubation with 1 μg/ml Hoechst 33342 for 15 min at RT. Oocytes were finally rinsed in PBS/PVA and placed on a slide containing mounting solution, covered with a coverslip, and observed with a fluorescence microscope (Olympus BX51, Tokyo, Japan). The images were captured through a sensitive camera (Olympus DP71). The fluorescence intensity was assessed by Image J software (National Institutes of Health, Bethesda, MD). DNMT3a assessment was performed in triplicates. Antibodies characteristics are listed in supplementary information (Table S2).

### Immunofluorescence staining for detection of global DNA methylation

The global DNA methylation in matured oocytes, zygotes (18-h post insemination: hpi) and blastocysts (at day 7) was assessed using immunostaining through measuring the fluorescence intensity of the complexes between primary and secondary antibodies as described previously^53^. The procedure of immunostaining was exactly similar to the immunostaining of DNMT3a except for denaturation of DNA which was carried out with HCl (4 N) for 30 min after permeabilization process. After treating with HCl, oocytes/zygotes/blastocysts were directly treated with 0.1 M Tris–HCL (pH 9) for 20 min. Mouse 5-methylcytosine (5-mC) antibody (Eurogentec BI-MECY-0500, Belgium) and goat anti-mouse IgG conjugated with FITC (Millipore; AP124F) were used as the primary and secondary antibodies, respectively. Antibodies characteristics are listed in supplementary information (Table S2).

### Differential staining

The quality of the blastocysts was evaluated by differential staining allowing to distinguish and quantify the inner cell mass from the trophectoderm that appeared, respectively, pink or blue under UV light. Briefly, Day 8 blastocysts were washed in HTCM and 5 mg/ml BSA. After that the blastocysts were exposed to 0.5% Triton X-100 for 20 s and subsequently to 30 mg/ml propidium iodide for 30 s. At the end, embryos were stained and fixed for 15 min in cold solution (4 °C) consisting of ethanol and 10 mg/ml Hoechst. After mounting on slide, the blastocysts were examined using a fluorescent microscope (Olympus BX51, Tokyo, Japan).

### Statistical analysis

Continuous variables were analyzed using GLM procedure after testing for normality (Shapiro–Wilk) using UNIVARIATE procedure. Tukey Studentized Range (HSD) test was used for pair-wise comparisons. If the assumptions of parametric tests were violated, data were analyzed using Kruskal–Wallis test. Data with discrete nature were analyzed using GENMOD procedure including logistic regression (log) as Link Function and Binomial as type of distribution in the model. The percentage of events were calculated using FREQ procedure. Data were presented as mean ± s.e.m. and percentage. P < 0.05 was considered significant in all analyses. All statistical analyses were conducted in SAS (SAS, Statistical Analysis System, 2012. User’s Guide, version 9.4. SAS Institute, Cary, NC.).

## Supplementary Information

Supplementary Tables.

## Data Availability

The datasets used and/or analyzed during the current study available from the corresponding author on reasonable request.
