# Supplementary material for: Metabolic enhancement of the one carbon metabolism (OCM) in bovine oocytes IVM increases the blastocyst rate: evidences for a OCM checkpoint
Source: Sci Rep. 2022 Nov 30;12:20629. doi: 10.1038/s41598-022-25083-8 (PMC9712338; doi:10.1038/s41598-022-25083-8)
Supplement: Supplementary file 1 — Supplementary Tables. [file 41598_2022_25083_MOESM1_ESM.pdf]

Supplementary Table S1: List of primers used in this study for real time PCR

Genes	Primer sequences (5'–3')	Accession no.	TA (°C)	Product size
CBS	Forward: 5`-AGTTGGAGTGGCTGGAGTG-3`<br>Reverse: 5`-TTGAGAGGCGGATGAAAGGTT-3`	NM_001102000.2	54	156
MTR	Forward: 5`-TCGCTCACTCTCATCATTCC-3`<br>Reverse: 5`-GACCTAACCTCTACCATCATCC-3`	XM_010820411.3	52	139
BHMT	Forward: 5`-CAGACCTTCACCTTCTATGC-3`<br>Reverse: 5`-CTCCTTCATCAGCCACTTG-3`	NM_001011679.1	56	130
TNFAIP6	Forward: 5`- CTCACGGATGGGGATTCAA -3`<br>Reverse: 5`- ACCGCCTTCGCTTCTGC-3`	NM_001007813.2	61	127
HAS2	Forward: 5`- ATGGTTGGAGGTGTCG-3`<br>Reverse: 5`- AGAGGTCCGCTAATGC -3`	NM_174079.3	58	152
DNMT1	Forward: 5`- GAAGCAGAATAAGAATCGG -3`<br>Reverse: 5`- TTTGAAGAGTCGTCTGGAA -3`	NM_182651.2	54	144
DNMT3A	Forward: 5`- TGGTCCTGGGCGTTAG -3`<br>Reverse: 5`- CCTGCTTTATGGAGTTCG -3`	NM_001206502.2	57	252
POU5F1	Forward: 5`- GGAAAGGTGTTCAGCCA -3`<br>Reverse: 5`- ATTCTCGTTGTTGTCAGC -3`	NM_174580.3	57	123
NANOG	Forward: 5`- TTGTGACGGCTATTGTATG -3`<br>Reverse: 5`- ACCTCTTACTGGACTCATT -3`	NM_001025344.1	53	159
TEAD4	Forward: 5`- AAAGTGGAGACCGAGTAT -3`<br>Reverse: 5`- GCTTGTGGATGAAGTTGA -3`	XM_010805630.3	52	100
B actin	Forward: 5`- TTCCTGGGTATGGATCCTG -3`<br>Reverse: 5`- GGTGATCTCCTTCTGCATCC -3`	XM_015467124.1	58	130

Supplementary Table S2: List of antibodies used for immunofluorescence flowcytometry.

Antibody	Supplier	Catalog number	Species	Type	Dilution	References
DNMT3A (64B1446) protein antibody (primary antibody)	Novus	NB120-13888	Mouse	Monoclonal/ IgG1 Kappa	1/200	
Mouse 5-methylcytosine (5-mC) antibody (Primary antibody)	Eurogentec	BI-MECY-0500	Mouse	Monoclonal/IgG	1/400	[1-3]
Goat Anti-Mouse IgG antibody, FITC conjugated (Secondary antibody)	Millipore	AP124F	Goat	Polyclonal/IgG	1/200	[1-3]

References:

1 Alsalim, H., Jafarpour, F., Tanhaei Vash, N., Nasr-Esfahani, M. H. & Niasari-Naslaji, A. Effect of DNA and histone methyl transferase inhibitors on outcomes of buffalo–bovine interspecies somatic cell nuclear transfer. *Cellular reprogramming* **20**, 256-267 (2018).

2 Jafari, S. *et al.* Epigenetic modification does not determine the time of POU5F1 transcription activation in cloned bovine embryos. *Journal of assisted reproduction and genetics* **28**, 1119-1127 (2011).

3 Jozi, M. *et al.* Induced DnA hypomethylation by folic Acid Deprivation in Bovine fibroblast Donor cells improves Reprogramming of Somatic cell nuclear transfer embryos. *Scientific reports* **10**, 1-13 (2020).
